# Detection of *Mycobacterium bovis* in Organs of Slaughtered Cattle by DNA-Based Polymerase Chain Reaction and Ziehl-Neelsen Techniques in Bauchi State, Nigeria

**DOI:** 10.1155/2015/921868

**Published:** 2015-02-03

**Authors:** A. S. Sa'idu, E. C. Okolocha, A. A. Dzikwi, J. K. P. Kwaga, A. A. Gamawa, A. Usman, S. A. Maigari, S. Ibrahim

**Affiliations:** ^1^Department of Veterinary Public Health and Preventive Medicine, Ahmadu Bello University, PMB 1013, Zaria 2222, Kaduna State, Nigeria; ^2^Area Veterinary Clinic (Kofar Ran), Ministry of Animal Resources and Nomadic Resettlement, Bauchi State, Nigeria; ^3^TB-Laboratory, Department of Medicine, Faculty of Veterinary Medicine, Ahmadu Bello University, PMB 1013, Zaria 2222, Kaduna State, Nigeria; ^4^Department of Medicine Faculty of Veterinary Medicine, Ahmadu Bello University, PMB 1013, Zaria 2222, Kaduna State, Nigeria; ^5^University of Maiduguri Teaching Hospital, PMB 1069, Maiduguri, Borno State, Nigeria

## Abstract

Bovine tuberculosis is a chronic, infectious, and contagious zoonotic disease of domestic animals, wild animals, and humans. It also poses a public health threat and economic losses. This study was aimed at determining the prevalence of bovine tuberculosis in slaughtered cattle, based on PM meat inspection, Ziehl-Neelsen staining, and PCR techniques in Bauchi State, Nigeria. A Prospective study was conducted on 800 cattle slaughtered in the three Zonal abattoirs of Bauchi State, Nigeria. One hundred and twenty (15%) tissues from different organs had suspected bTB lesions at PM. Out of the samples examined 35 (29.2%) were AFB positive by ZN and 10 (8.3%) were confirmed positive for* M. bovis *by PCR, with an overall prevalence of 29.16% and 8.33%, respectively. Female had a higher prevalence rate than male cattle at 16.66% and 12.5 % by ZN and 5.00% and 3.33% by PCR, respectively (*P*>0.05, *χ* ^2^ = 0.218). However, there was a statistically significant association (*P*<0.05, *χ* ^2^ = 7.002) between detection of bTB and the age of cattle. ZN revealed that cattle aged 6 years and above had the highest number of positive bTB cases 67.9%, while cattle aged 3–5 years had the lowest 14.81%. PCR technique revealed that the cattle aged 6 and above years also had the highest percentage positive* M. bovis *cases of 22.84%, whereas cattle aged 3–5 years had the lowest and the overall prevalence rate of 8.33%. The study found a high infection rate of bTB among cattle and majority of the lesions 54.2% were from lungs. The prevalence of bTB was higher in Bauchi metropolitan abattoir which supplies larger population of the state with beef.

## 1. Introduction

Bovine tuberculosis (bTB) is a chronic infectious and contagious zoonotic disease of domestic animals, wild animals, and humans [1]. It also occurs in a wide range of mammalian species [2]. It is characterized by the formation of granulomas in tissues especially in the lungs, lymph nodes, liver, intestines, and kidney [3]. According to the World Health Organization report in 2013, tuberculosis still remains a major health problem with 9 million new cases and 1.5 million deaths annually worldwide [4]. The majority of these occur in the developing nations. In Nigeria, there have been limited studies to determine the prevalence/relationship between bovine and human TB especially with the emerging culture of eating improperly cooked beef and mutton, along with the drinking of unpasteurized fresh milk [5, 6].

Bovine tuberculosis is caused by* Mycobacterium bovis *(bovine tubercle bacillus) which is a member of* Mycobacterium tuberculosis* complex [7, 8]. The aetiological agents of mammalian tuberculosis, classified as members of the* Mycobacterium tuberculosis* complex (MTBC), include* Mycobacterium tuberculosis, M. bovis, M. microti, M. caprae, M. africanum, M. canettii, *and* M. pinnipedii*.* Mycobacterium africanum *consists of a rather heterogeneous group of strains isolated from humans in Africa [9]. The organism may be transmitted by aerosol or droplets of exudates containing the bacilli. It can be transmitted by ingestion of feed and water contaminated with urine, fecal material, or exudates from diseased animals that contain the tubercle bacilli. The bovine tubercle bacillus is the cause of the bTB in cattle, but still is often used to denote bovine strains of the tubercle bacillus irrespective of the host. In fact, the bovine tubercle bacillus has one of the broadest host ranges of all known pathogens. The species has been reported in domesticated and feral Bovidae. Other species in which the disease has been reported include goat, sheep, pig, horse, cat, dog, fennec fox, bison, buffalo, badger, wild and feral pig, antelope, camel, and man and nonhuman primates, among others [10]. Cattle movements, particularly those from areas where bTB is reported, are the best predictor of disease occurrence [11].

Bovine TB in cattle is initially asymptomatic and, prior to testing, farmers usually remain unaware of the outbreak during this stage. It can take as long as a few years before any signs can be observed. As the infection progresses in cattle, bovine tuberculosis causes a slight fever and roughened hair coat and the lymph nodes usually swell or have abscesses and show signs of dyspnoea with an associated cough. The cow will appear weak, show signs of laboured breathing, and have an increased breathing rate. A loss of appetite will be noticed and the decrease in weight may be so significant that the cow actually becomes emaciated. Tuberculosis (TB) in humans is characterized by prolonged coughing, general body weakness, wasting, and sweating at night with slight fever [12].

In UK, badgers have been culled since 1971, when a tuberculous dead badger was found in an infected farm. Bovine TB control has proved difficult to achieve where there is a persistent reservoir of infection in wildlife [13]. Consequently, interpreting subsequent trends in incidence of bTB which are complicated by multiple factors such as cattle management practice, changing specificity of tests, changes to the cattle meat factory (abattoir) including pollution control/regulations, and increase in level of aflatoxins in feeds (known for their immunosuppressive qualities, etc.,) [14, 15].

Despite the availability of effective therapy, tuberculosis remains a major public health problem worldwide [16, 17]. Factors such as inadequate health service infrastructure, decreased access to health care, and limited human and financial resources have prevented adequate implementation of control measures against TB [18].

Recent advances in the treatment of human TB have permitted effective management of cases on an ambulatory basis [19]. However, in many developing countries, an irregular drug taking practice and premature termination of treatment mainly by self-discharge are the main cause of poor performance of control programmes. This study was aimed at determining the prevalence of bovine tuberculosis in slaughtered cattle, based on postmortem (PM) meat inspection, Ziehl-Neelsen staining (ZN), and polymerase chain reaction (PCR) techniques in abattoirs of Bauchi State Nigeria.

## 2. Materials and Methods

### 2.1. Study Area

Bauchi State occupies a total land area of 49,119 Km² representing about 5.3% of Nigeria's total land mass and is located between latitudes 9°3′ and 12°3′ north of the equator and longitudes 8°50′ and 11° east of the Greenwich Meridean [20]. The state is bordered by Kano and Jigawa to the north, Taraba and Plateau to the south, Gombe and Yobe to the east, and Kaduna to the west. The state is highly populated with cattle mainly owned by Fulani herdsmen. The cattle population is estimated at 1,789,000, about 13% of the Nigerian cattle population of 13,900,000 [21]. The State has a human population of 4,653,066; ranked 11th of the 36 states, density of 95 km^2^ (250/sqmi) and per capita income of $983 [22]. Bauchi State has a total of 55 tribal groups in which Hausa, Fulani, and Kanuri are the main tribes. The study was carried out in Bauchi, Katagum, and Misau Local Government Areas (out of the 20 LGAs), each representing the three senatorial zones as Bauchi South, Bauchi North, and Bauchi Central with populations of 493,810, 295,970, and 263,487, respectively.

### 2.2. Study Design

The three major slaughter houses, namely, Bauchi, Misau, and Katagum (Azare), in each of the three senatorial zones of Bauchi State were selected for the study for sampling purposes, based on their daily slaughter capacity and socioeconomic impact of the abattoir. Accordingly, out of the 800 head of cattle examined (sample size), a proportionate distribution was employed, given 50% (400) to Bauchi abattoir and 25% (200), each, to Katagum and Misau slaughter houses. This was based on their daily slaughter capacity of 40–60, 20–30, and 20–25 head of cattle from Bauchi abattoir, Katagum, and Misau slaughter houses, respectively. The selection of the cattle sampled at each of the abattoirs was strictly on the basis of the age of the cattle (meant for slaughter and three years above), following then PM observation of typical granulomatous bTB lesions in any of the visceral organs before the cattle is considered a case and sample.

### 2.3. Source of Animals for Slaughter

Most of the animals brought for slaughter were mainly sourced from Maiduguri, Chad, and Niger.

### 2.4. Sampling Procedure and Transportation

Identification of cases was based on presence of typical tubercle, yellowish, granulomatous, and caseous lesions in the lungs, lymph nodes, kidneys, intestines, and liver [23]. Aging of cattle was carried out at the abattoir after slaughter as described by [24], using the time of appearance and the degree of wear on the temporary and permanent teeth. Additional data taken included sex and breed of each animal sampled.

A total of 800 head of slaughtered cattle were examined at postmortem for TB lesions. Using a convenience sampling, a total of 400 head of cattle from Bauchi, 200 head of cattle each from Misau and Katagum LGA slaughter houses were examined. One hundred and twenty (120) tissue samples were collected from cattle with suspected TB lesions into sterile screw-capped containers (with 9% normal saline solution to keep tissues moist) and transported inside a cooler containing ice pack to the Veterinary Public Health Bacterial Zoonoses Laboratory, Ahmadu Bello University, Zaria, and stored at −20°C until analysis was carried out. Stored samples were processed (crushed and analysed) for microscopic examination (modified Ziehl-Neelsen staining technique) and only AFB positive samples were taken to the DNA Labs, Kaduna, and used for conventional PCR amplification protocols specific for* M. bovis*, as described by [25].

## 3. Microscopic Examination

### 3.1. Acid Fastness/Ziehl-Neelsen Staining Technique

Ziehl-Neelsen staining (“Hot” ZN technique) was carried out on the tissues stored at −20°C after thawing them to room temperature using standard protocol for mycobacterial identification of [26] to detect acid fast bacilli from granulomatous tissue samples collected during the meat inspection.

Direct smears were prepared from tissues presenting tuberculous lesions using Ziehl-Neelsen staining/acid fast technique. The collected samples were crushed using a pestle in a clean mortar and homogenized using a stomacher. The crushed tissues were placed on a new, clean, and labelled grease-free glass slide and spread with a sterile pipette tip to make a smear (1 cm × 2 cm). The smeared slides were air-dried and heat-fixed by passing them gently over the flame (Bunsen burner) with the specimen side up. This fixed the specimen to the slide and preserved the bacterial morphology.

### 3.2. Protocol for Hot Ziehl-Neelsen

Direct smears were prepared. The crushed tissues were placed on a grease-free glass slide to make a smear (1 cm × 2 cm). The smears were then stained with carbol fuchsin and allowed to stand for 5 minutes. The stained smear was washed with tap water (to remove excess stain from the slide). The slide was decolourized with 3% acid alcohol. After decolourization, the slide was counterstained with methylene blue for 1 minute. Additional rinsing was done with water to remove excess colour and the slide was allowed to air-dry. Finally, the slide was viewed in an optical microscope (×100—an oil emersion objective). Results were that acid fast bacilli appeared red. A red, straight or slightly curved rod occurring singly or in a cluster indicates the presence of tubercle bacilli.

### 3.3. Genomic DNA Preparation from Tissues

The tissue samples positive by ZN were collected into a 1.5 mL microfuge tube containing lysis buffer, stored at room temperature, and transported on ice pack to DNA Labs, Kaduna. About 1 g of the tissue sample was homogenized with pestle and mortar for chromosomal DNA extraction using a phenol-chloroform technique as described by [25, 27]. The supernatant was discarded; the suspension was used, then cooled at 4°C, neutralized with 3 volumes of 0.1 m Tris-HCL (PH. 7.4) buffer, and centrifuged (5,000 ×g, 5 min) to get rid of the tissue membrane and possible contaminants. The pellet was dried and redissolved in 20 *μ*L of 1x Tris-Borate (1× TBE) buffer and DNA was precipitated with ethanol, collected by centrifugation and dissolved in 50 *μ*L of distilled water. 5 *μ*L of the extracted DNA was run on 1.0% agarose gel and spectrophotometer to confirm the presence of DNA. The remaining DNA samples were stored at −20°C until further use.

Table 1 showed the forward and reverse oligonucleotide primer sequences that were complimentary to the DNA template extracts at the 3′ ends in the nucleotide sequences of the* Mycobacterium bovis* genome. The primers were synthesized based on the G+C contents of the* Mycobacterium bovis* strains available at the gene bank of the Bioneer, USA [28].

### 3.4. PCR Amplification Protocols

Ten (10) microlitres suspended DNA was used as a template for PCR amplification under standard conditions as described by [29]. A commercial “Hot-Stat” PCR Premix (Bioneer, USA), a mixture prepared in a lyophilized format containing Taq DNA polymerase, reaction buffer, dNTPs (dATP, dGTP, dCTP, dTTP), and MgCl_2_ were used. All amplification reactions were performed using a Perkin Elmer Thermocycler (Perkin Elmer Cetus) programmed for 40 amplification cycles [28, 30]. The reaction was performed in a final volume of 50 *μ*L containing 10 *μ*L of DNA template, 1x TBE (1x Tris-Borate) reaction buffer (containing 10 mM Tris-HCl (pH 8.3), 50 mM KCl, 1.3 mM MgCl_2_, and 0.001% of gelatin), 2.5 U Taq polymerase, 0.2 mM of each deoxynucleoside triphosphate, and 75 pmol of each primer (Table 1). DNA from* M. bovis* ATCC 19210 and BCG Pasteur 27291 and sterile nuclease-free water were used as positive and negative PCR controls, respectively. After an initial denaturation step (at 94°C, for 5 min), 40 amplification cycles were performed as follows: denaturation at 94°C for 1 min, annealing at 58°C for 30 sec, and extension at 72°C for 30 sec, with an increment of 1 sec per cycle for the denaturation and extension segment. A final extension was performed at 72°C for 15 min.

### 3.5. Gel Electrophoresis

After amplification, 20 *µ*L of the PCR product was loaded with gel loading buffer in a 1% agarose gel (Swekem; FMC Bioproducts, Rockland, Maine, USA) containing 0.5 pug/mL ethidium bromide. The gel was also loaded with the 100 base pair (bp) DNA molecular marker (ladder). Gel electrophoresis was done at a 5 v/cm for 1 hour. Finally, after electrophoresis, the DNA bands were visualized (using UV light box or gel imaging system) and photographed.

### 3.6. Data Analysis

Descriptive statistical tables and bar charts were constructed using Microsoft Excel 2010. The data were also analysed using statistical package for social sciences (SPSS) version 20.0. Chi-square (*χ* ^2^) was used to determine association between variables and* M. bovis *infection. Odds ratio (OR) and 95% confidence interval were calculated to measure the strengths of associations between variables and bTB (*M. bovis*). Values of *P* < 0.05 were considered significant.

## 4. Results

**Figure 1:**
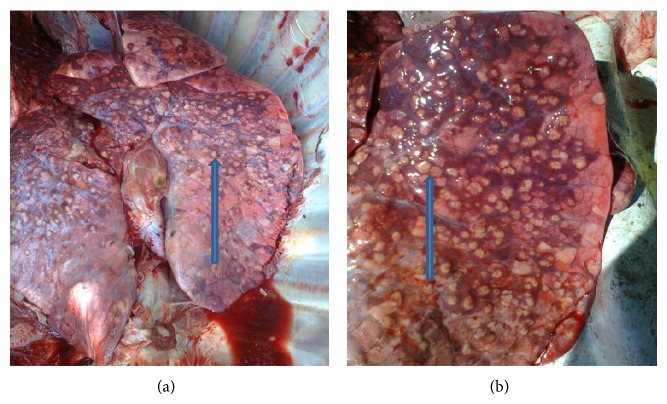
Photograph of the affected lungs showing massive granulomatous (tuberculous) lesions from slaughtered cattle, during PM ((a) bilateral lobes and (b) unilateral lobe). See the blue arrows.

**Figure 2:**
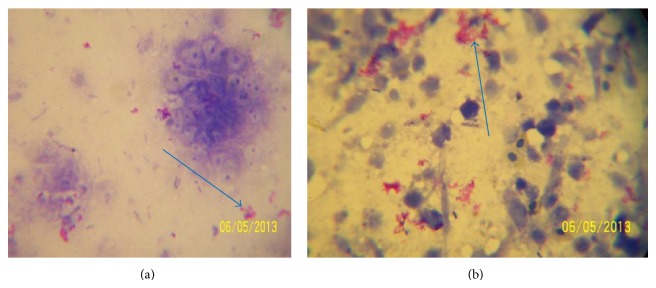
Photographs (a) and (b) showing the acid fast bacilli (AFB) organisms appearing reddish under a microscope (×100 oil immersion), pointed by the arrows on the methylene blue background after the Ziehl-Neelsen (ZN) staining.

**Figure 3:**
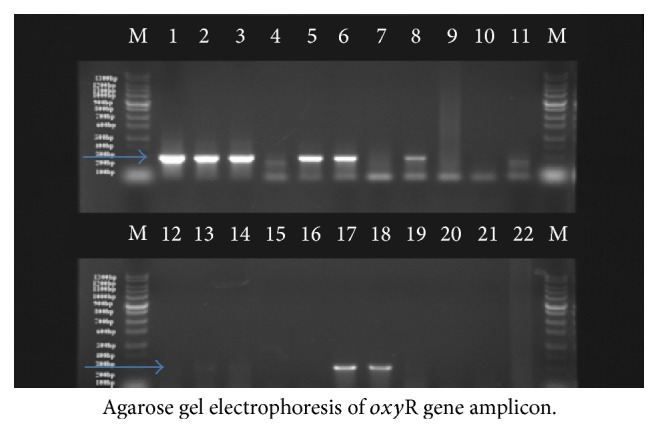
A 2-panel agarose gel electrophoresis of PCR amplification of* oxy*R* gene* specific for* M. bovis*. Lane M 1.3 kb molecular weight markers (100 bp DNA ladder, Bioneer labs, USA); lanes 1 and 2 are positive controls (BCG Pasteur strain and BCG vaccine), lane 10 is negative control (nuclease free water), and lanes 3, 5, 6, 8, 17, and 18 are positive samples diagnostic for* M. bovis *(285 bp).

Of the 800 samples examined at postmortem for bTB lesions, only 15% (120) tissues from different organs were suspected to have bTB lesions. Out of the 120 tissues, thirty-five 29.16% (35) were positive for bTB using Ziehl-Neelsen staining technique (Figure 2) and gave a prevalence rate of 29.16% (Tables 3 and 4). Further confirmation using PCR technique (Figure 3) gave an overall prevalence of 8.33% (10). The occurrence of bTB lesions in the organs of slaughtered cattle in Bauchi State showed that the lungs had the highest number of bTB suspected tissues 54.20% (65/120); Figure 1 showed a clear photograph of tuberculous lung lesions obtained from affected cattle at PM. Followed by the lymph nodes 23.30% (28/120) while the heart, liver, spleen, intestines and mammary glands made up the other 22.50% (27/120) suspected bTB tissues (Table 2). By ZN microscopic staining 29.23% (19/65) of the lungs were positive for bTB (AFB), while 21.43% (6/28) of the lymph nodes were AFB positive, followed by the heart, the intestines, the liver, and the spleen with the few AFB positive and mammary gland was AFB negative. More so, by PCR, the lungs also had significantly higher number of positive results for* M. bovis*, 7.7% (5/65), followed by the lymph nodes 7.14% (2/28) and then the intestines and the liver had the least number of positives for bTB (*M. bovis*), while the spleen, heart, and mammary gland were all negative for bTB (Table 2).

There was a statistically significant (*P* < 0.05; *χ* ^2^ = 4.623, OR = 3.680) association between detection of the bTB in the tissues using ZN and the age of cattle (Table 2). Cattle aged 6 years and above had the highest percentage tissue positive for bTB 33.33% (31/93) using ZN, while cattle aged 3–5 years had 14.81% (4/27) of their tissues positive for bTB and a prevalence rate of 29.16%, for the three age groups. The PCR also showed a statistically significant (*P* < 0.05; OR = 3.363) association between detection of bTB in the tissues and the age of the cattle. Cattle aged six (6) years had a prevalence rate of 8.33%, while cattle aged 3–5 years were negative for bTB using PCR method.

There was no statistically significant association (*P* > 0.05; *χ* ^2^ = 2.017, OR = 1.80) between the presence of bTB in the tissues sampled and the sex of the cattle using ZN method. However, the female cattle had higher prevalence and sex-specific rates than the male cattle sampled (Table 5). The male cattle also had higher sex-specific rate of 10% (4/40) using PCR technique than the female cattle, 7.50% (6/80). However, female cattle had higher prevalence rate of 5.00% than male cattle with 3.33%, and there was no statistically significant association between detection of bTB and sex, using PCR method (*χ* ^2^ = 0.218, df = 1, OR = 1.37, 95% CI on OR = 0.364–5.164) (Table 5).

Table 5 also summarises the overall prevalence and sex-specific rates using ZN and PCR tests. Using ZN, the prevalence and sex-specific rates of 12.5% (15/120) and 37.50% (15/40), respectively, out of the male cattle sampled were AFB positive while 16.66% (20/120) out of the female cattle population, 25.0% (20/80), respectively, were also AFB positive, given an overall prevalence of 29.16% by ZN, whereas, using PCR, the prevalence and sex-specific rates of the cattle population sampled indicated a rate of 8.33% (10/120) out of the 40 head of male cattle sampled, 10.0% (4/40), while out of the 80 head of female cattle sampled, 7.50% (6/80) were positive for bTB specifically with* M. bovis*. The infection rates were higher in females than in males. By the confirmatory PCR, the overall prevalence rate of* M. bovis* (bTB) in Bauchi State was 8.33%.

## 5. Discussion

The prevalence rates of bTB found in this present study based on ZN and confirmatory PCR were 29.16% and 8.33%, respectively. This study also showed that PCR is a highly sensitive and specific (8.33% and 90.1%, resp.) test that can be adapted as a confirmatory test to conventional tests such as PM, tuberculin skin test (TST), and ZN because of its ability to rule out possible false positive results associated with these tests. However, ZN has the ability to detect more AFB positive samples as fastidious organisms that may be present in the granulomatous lesions obtained from slaughtered cattle. This agreed with the previous report by [31] using ZN method alone. The overall prevalence rate (8.33%) of this study is relatively high and may be related to the poor control measures in the state (lack of test and slaughter policy, absence of control at borders, inadequate quarantine measures, and the lack of effective preventive measures against bovine TB) and the influx of possibly infected cattle from neighbouring states and countries (Cameroon, Chad, and Niger). Also the increase in intensive farming practice where large herds are housed together for long periods of time and poor hygiene are possible risk factors to the spread and endemicity nature of the disease in the state. There are many studies on bTB in Nigeria and other countries including a retrospective study by [32] who reported a relatively lower prevalence of 1.72% in Bauchi State as compared to our findings, which may be due to the improved diagnostic technique used in this study. Moreover, increase in free influx of possibly infected cattle from neighbouring endemic states, especially, Gombe State, with a prevalence rate of 12.27% as reported by [32], who described Bauchi State as a population at risk, could also be a reason for the high prevalence reported in this study. Also a high prevalence rate of the disease in the Northern Nigeria and increase in livestock density and contact rates among cattle of different sources could be another reason.

The findings that 120 (15%) bTB suspected lesions were observed in the 800 head of slaughtered cattle examined in Bauchi State abattoirs had emphasized the importance of PM meat inspection. This agrees with the report of [33], as postmortem examination still remains the immediate diagnostic tool to be used in endemic slaughter houses.

The distribution and occurrence of suspected bTB lesions in different organs of slaughtered cattle in Bauchi State showed 54.20% (65/120) in the lungs and 23.30% (28/120) in the lymph nodes, while the heart, liver, spleen, intestines, and mammary glands made up the least number of bTB positives. This agreed with the retrospective study reported by [33] on gross bTB lesions in different organs in slaughtered cattle in Maiduguri, Nigeria, with 67.8% of the lungs and 13.9% lymph nodes, with the least number in the other affected visceral organs. This also agreed with the report of [34].

## 6. Conclusion

The present study estimated the prevalence rate of bTB in Bauchi State, using PM, ZN, and PCR techniques at (15.0%, 29.16%, and 8.33%, resp.). Bovine TB lesions found at PM were not all due to* M. bovis *alone, as other MTBC and AFB organisms may cause bTB-like lesions which were excluded by PCR.

## 7. Recommendation

Proper PM meat inspection should be practiced efficiently at the abattoir and slaughter houses, before taking beef to the public. The emergence MDR and XDR-TB strains are also a major concern in Nigeria. Thus, further molecular epidemiological studies with more improved techniques, like MIRU-VNTR and spoligotyping, should be carried out on isolates from the state to look for other zoonotics, like* M. africanum.*

## Figures and Tables

**Table 1:** Species-specific primers for *Mycobacterium bovis* used for the study.

Primer direction	PCR primer sequence	Amplicon size (bp)
Forward	(5′CCCGCTGATGCAAGTGCC3′)	285 bp *M*. *bovis *
Reverse	(5′CCCGCACATCCCAACACC3′)	

These primer oligonucleotide sequences were used to amplify a fragment of the *oxy*R gene [29].

**Table 2:** Distribution of suspected bTB lesions in various organs of slaughtered cattle in Bauchi state, Nigeria.

Organs examined	Number of TB suspected lesions (%)	ZN staining (% +ve)	PCR (% +ve)
Lungs	65 (54.20)	19 (29.23%)	5 (7.80%)
Lymph nodes	28 (23.30)	6 (21.43%)	2 (7.14%)
Intestines	2 (1.70)	2 (100%)	2 (100%)
Liver	8 (6.70)	2 (25%)	1 (12.50%)
Spleen	6 (5.00)	1 (16.67%)	0 (0%)
Heart	10 (8.30)	5 (50%)	0 (0%)
Mam. gland	1 (0.80)	0 (0%)	0 (0%)
Total	**120**	**35**	**10**

**Table 3:** Distribution of bTB among cattle of different age groups in Bauchi State, Nigeria.

Age (years)	Number sampled	Number of ZN positive (%)	Prevalence	OR (95% CI)
3–5	27	4 (14.81)	3.33	1
6–8	41	16 (39.02)	13.33	3.680 (1.072–12.630)^*^
9–11	52	15 (28.85)	12.50	2.331 (0.688–7.892)
Total	**120**	**35**	**29.16**	

^*^Significant at 95% CI; *χ* ^2^ = 4.623; df = 2.

**Table 4:** Overall prevalence of bTB (*M*. *bovis*) among various age groups of cattle in Bauchi State, Nigeria.

Age (years)	Number sampled	PCR-positive (%)	Prevalence (%)	OR	95% CI
3–5	27	0 (0.00)	0.00		1^**^
6–8	41	7 (17.07)	5.83	3.363^*^	0.812–13.930
9–11	52	3 (5.77)	2.50		
Total	**120**	**10 **	**8.33**		

*χ* ^2^ = 7.002; ^*^ *P* < 0.05; df = 2; ^**^reference value (1).

**Table 5:** The overall prevalence and sex-specific rates of bTB in Bauchi State, Nigeria.

Sex^**^	Tests			Overall prevalence (%)	
	ZN		PCR	ZN^*^	PCR
	Number sampled	Positive (%)	Positive (%)		
Males	40	15 (37.50)	4 (10.00)	12.50	3.33
Females	80	20 (25.00)	6 (7.50)	16.66	5.00
Total	**120**	**35**	**10**	**29.16**	**8.33**

^*^ *χ* ^2^ = 17.09, df = 1, OR = 4.53, *P* < 0.05, and 95% CI = 2.12–9.664.

^*^Significant between tests.

^**^ *χ* ^2^ = 0.218, OR = 1.37.

^**^Between sexes.

## References

[1] Radostits O. M., Blood D., Hinchey K. (2007). *Veterinary Medicine: A Textbook of the Diseases of Cattle, Horses, Sheep, Pigs and Goats*.

[2] O'Reilly L. M., Daborn C. J. (1995). The epidemiology of *Mycobacterium bovis* infections in animals and man: a review. *Tubercle and Lung Disease*.

[3] Shitaye J. E., Tsegaye W., Pavlik I. (2007). Bovine tuberculosis infection in animal and human populations in Ethiopia: a review. *Veterinarni Medicina*.

[4] WHO (2014). World Health Organization global TB report (2014): Tuberculosis (TB). *Fact sheet*.

[5] Caffrey J. P. (1994). Status of bovine tuberculosis eradication programmes in Europe. *Veterinary Microbiology*.

[6] Shehu L. M. (1988). *Survey of tuberculosis and tubercle bacilli in Fulani herds, “Nono” and some herdsmen in Zaria area, Nigeria [M.S. thesis]*.

[7] Collins C. H., Grange J. M. (1983). The bovine tubercle bacillus. *Journal of Applied Bacteriology*.

[8] Pfeiffer U., Deviewa P. D. (2003). Tuberculosis in animals. *Clinical Tuberculosis*.

[9] Collins C. H., Grange J. M. (1987). Zoonotic implication of *Mycobacterium bovis* infection. *International Veterinary Journal*.

[10] Francis J. (1958). *Tuberculosis in Animals and Man*.

[11] Gilbert M., Mitchell A., Bourn D., Mawdsley J., Clifton-Hadley R., Wint W. (2005). Cattle movements and bovine tuberculosis in Great Britain. *Nature*.

[12] Thoen C. O., Lobue P. A., Enarson D. A., Kaneene J. B., de Kantor I. N. (2009). Tuberculosis: a re-emerging disease in animals and humans. *Veterinaria Italiana*.

[13] Morris R. S., Pfeiffer D. U., Jackson R. (1994). The epidemiology of *Mycobacterium bovis* infections. *Veterinary Microbiology*.

[14] Clifton-Hadley R. S., Wilesmith J. W., Richards M. S., Upton P., Johnston S. (1995). The occurrence of Mycobacterium bovis in cattle in and around an area subject to extensive badger (Males males) control. *Epidemiology and infection*.

[15] FAWC (1997). Farm Animal Welfare Council/annual report. *FAWC*.

[16] Raviglione M. C., Snider D. E., Kochi A. (1995). Global epidemiology of tuberculosis: morbidity and mortality of a worldwide epidemic. *Journal of the American Medical Association*.

[17] Wada T., Maeda S., Hase A., Kobayashi K. (2007). Evaluation of variable numbers of tandem repeat as molecular epidemiological markers of *Mycobacterium tuberculosis* in Japan. *Journal of Medical Microbiology*.

[18] Maher D., Van Gorkom J. L. C., Gondrie P. C. F. M., Raviglione M. (1999). Community contribution to tuberculosis care in countries with high tuberculosis prevalence: past, present and future. *International Journal of Tuberculosis and Lung Disease*.

[19] Morisky D. E., Malotte C. K., Choi P. (1990). A patient education program to improve adherence rates with antituberculosis drug regimens. *Health Education Quarterly*.

[20] Bauchi State Diary http//:www.nigeriagalleria.com/nigeria/statenigeria/bauchistate.

[21] FAO (2010). Corporate documentary repository. *Nigerian Cattle Population*.

[22] http://www.nigeriamasterweb.com/Nigeria06CensusFigs.html.

[23] Corner L., Melville L., McCubbin K. (1990). Efficiency of inspection procedures for the detection of tuberculous lesions in cattle. *Australian Veterinary Journal*.

[24] Ron T., Ben B., Bill K., Ken C. Methods of determining age in cattle.

[25] del Portillo P., Murillo L. A., Patarroyo M. E. (1991). Amplification of a species-specific DNA fragment of *Mycobacterium tuberculosis* and its possible use in diagnosis. *Journal of Clinical Microbiology*.

[26] Kazwala R. R., Daborn C. J., Sharp J. M., Kambarage D. M., Jiwa S. F. H., Mbembati N. A. (2001). Isolation of Mycobacterium bovis from human cases of cervical adenitis in Tanzania: a cause for concern?. *International Journal of Tuberculosis and Lung Disease*.

[27] Chomczynski P., Sacchi N. (1987). Single-step method of RNA isolation by acid guanidinium thiocyanate-phenol-chloroform extraction. *Analytical Biochemistry*.

[28] Rodriguez J. G., Mejia G. A., Del Portillo P., Patarroyo M. E., Murillo L. A. (1995). Species-specific identification of *Mycobacterium bovis* by PCR. *Microbiology*.

[29] Romero R. E., Garzón D. L., Mejía G. A., Monroy W., Patarroyo M. E., Murillo L. A. (1999). Identification of *Mycobacterium bovis* in bovine clinical samples by PCR species-specific primers. *Canadian Journal of Veterinary Research*.

[30] Mamiatis T., Fritsch E. F., Sambrook J. (1982). *Molecular Cloning: A Laboratory Manual*.

[31] Abubakar I. A. (2007). *Molecular epidemiology of human and bovine tuberculosis in the Federal Capital Territory and Kaduna state [Ph.D. thesis]*.

[32] Aliyu M. M., Adamu A. J., Bilyaminu Y. A. (2009). Current prevalence of tuberculous lesions among slaughtered cattle in northeastern state of Nigeria. *Revue Elevage Veterinaire pays des Tropicaux*.

[33] Abubakar U. B., Shehu S. A., Mohammed F. U. (2011). Retrospective study of tuberculosis in slaughtered cattle at Maiduguri abattoir, Nigeria. *Veterinary Research*.

[34] Igbokwe I. O., Madaki I. Y., Danburam S., Ameh J. A., Aliyu M. M., Nwosu C. O. (2001). Prevalence of pulmonary tuberculosis lesions in cattle slaughtered in abattoirs in Northeastern Nigeria. *Revue d'Elevage et de Médecine Vétérinaire des Pays Tropicaux*.

